# Spectral ultrahigh-resolution photon-counting CT for coronary stent imaging: evaluation in a dynamic phantom

**DOI:** 10.1186/s41747-025-00654-2

**Published:** 2025-12-02

**Authors:** Muhammad Taha Hagar, Tilman Emrich, Milán Vecsey-Nagy, Fabian Bamberg, Christopher L. Schlett, G. William Garrison, Ava Wenderoth, Alexander Isaak, Daniel Kuetting, Julian A. Luetkens, Constantin von Zur Mühlen, Akos Varga-Szemes, Dmitrij Kravchenko

**Affiliations:** 1 Department of Radiology and Radiological Science, Medical University of South Carolina, Charleston, SC USA; 2 Department of Diagnostic and Interventional Radiology, Medical Centre, Faculty of Medicine, University of Freiburg, Freiburg, Germany; 3 Department of Diagnostic and Interventional Radiology, University Medical Center of the Johannes Gutenberg-University, Mainz, Germany; 4 German Centre for Cardiovascular Research, Partner site Rhine-Main, Mainz, Germany; 5 Heart and Vascular Centre, Semmelweis University, Budapest, Hungary; 6 Department of Diagnostic and Interventional Radiology, University Hospital Bonn, Bonn, Germany; 7 Department of Cardiology, Medical Center University of Freiburg, and Faculty of Medicine, University of Freiburg, Freiburg, Germany

**Keywords:** Artifacts, Computed tomography angiography, Coronary vessels, Phantoms (imaging), Stents

## Abstract

**Background:**

We compared ultrahigh-resolution (UHR) photon-counting detector-computed tomography (PCD-CT) and spectral post-processed images for coronary stent visualization in a dynamic, anthropomorphic, and circulatory phantom.

**Materials and methods:**

Ten coronary stents were scanned at 60, 80, and 100 beats per min (bpm) using UHR-spectral PCD-CT (96 × 0.2 mm collimation). Reconstructions included UHR (0.2 mm), downsampled (0.6 mm), and spectral post-processed images (0.4 mm), including virtual monoenergetic images (VMI; 45–100 keV), lumen-preserving images, and iodine maps (IM). Objective quality was assessed by measurable stent lumen visibility and stent strut width overestimation factor, compared to nominal strut width. Subjective quality was rated using a 4-point Likert scale. Repeated-measures analysis of variance−ANOVA and Friedman test with *post hoc* corrections were applied.

**Results:**

UHR images provided the highest lumen visibility (62.6 ± 7.6%) at 60 bpm, outperforming all reconstructions ranging 43.5–52.7% (*p* ≤ 0.001) except IM (59.6 ± 11.9%, pairwise *p* = 0.839). UHR showed the lowest strut overestimation factor (18.1 ± 3.4), better than all spectral images (20.2–26.2, *p* ≤ 0.003) and DS (32.1 ± 6.5, *p* < 0.001). Subjective quality was best for UHR at 60 bpm (4.0 [interquartile range, IQR 4.0–4.0]) but declined at 100 bpm (3.0 [IQR 2.0–3.0], *p* < 0.01). VMI at 55 keV and IM maintained stable quality across heart rates (*p* ≥ 0.09).

**Conclusion:**

PCD-CT combining UHR and spectral imaging enhances stent assessment. UHR provides the best lumen visibility and strut accuracy but suffers from motion artifacts, whereas VMIs at 55 keV and IM remain stable across heart rates and potentially provide incremental value.

**Relevance statement:**

Combining UHR and spectral PCD-CT enhances coronary stent visualization by balancing high spatial detail with artifact reduction, potentially improving diagnostic confidence and enabling more reliable non-invasive follow-up across a range of heart rates in clinical practice.

**Key Points:**

- The combined value of spectral UHR CT for stent imaging remains largely unexplored.
- UHR PCD-CT showed the highest lumen visibility and sharpest strut delineation, whilst being prone to motion artifacts.
- Spectral reconstructions complement UHR by reducing artifacts and stabilizing image quality, especially at higher heart rates.

**Graphical Abstract:**

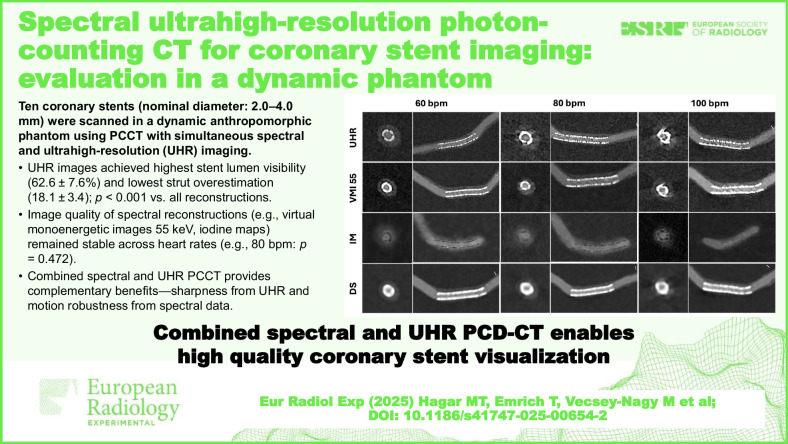

## Background

Recent international guidelines recommend coronary computed tomography (CT) angiography as the first-line test for patients with chest pain and low-to-intermediate risk [1–3]. However, its use in patients with known coronary artery disease, and especially with previous stent implantation, is only moderately supported [4]. Additionally, noninvasive stent imaging using a conventional energy-integrating detector (EID)-CT may only be considered as an alternative test for larger stents with an internal diameter of at least 3.0 mm [5]. This is due to photon-starvation and beam-hardening artifacts, mainly affected by partial volume averaging effects, which degrade image quality and hinder accurate noninvasive lumen assessment [6].

The clinical introduction of photon-counting detector (PCD)-CT technology in recent years has enabled significant advancements in cardiac and vascular imaging [7–10], particularly in the evaluation of coronary stents [11, 12]. Notably, the ability to visualize small anatomical structures at ultrahigh resolution (UHR) has yielded improvements in quantifying in-stent diameters [13], and enabled reliable performance in visualizing small-sized coronary stents *in vivo* and *in vitro* [14]. Moreover, PCDs provide spectral information due to their energy-resolving capability for individual x-ray photons, enabling the reconstruction of virtual monoenergetic images (VMI) and advanced spectral post-processing techniques, such as vessel lumen-preserving images and iodine maps (IM) [15, 16].

However, until the launch of the VB10 software version of the clinical dual-source PCD-CT scanner (see details below in the “Methods” section), acquiring electrocardiogram (ECG)-gated UHR images and utilizing advanced spectral capabilities were mutually exclusive, as both features could not be implemented concurrently. Recent iterations now enable the integration of both UHR and advanced spectral capabilities within a single acquisition [17]. This progress emphasizes the importance of evaluating optimal imaging strategies that balance UHR with the added value of spectral data in the context of coronary stent imaging. Specifically, it remains unclear whether polychromatic UHR images offer sufficient performance on their own or if the integration of spectral postprocessing, such as VMIs, lumen-preserving reconstructions, or IMs, provides incremental benefits. This drives the purpose of this investigation: to systematically compare UHR polychromatic PCD-CT and spectral postprocessed images for coronary stent visualization in a dynamic anthropomorphic phantom and to evaluate the potential added value of combining these approaches.

## Materials and methods

### Phantom setup

**Fig. 1:**
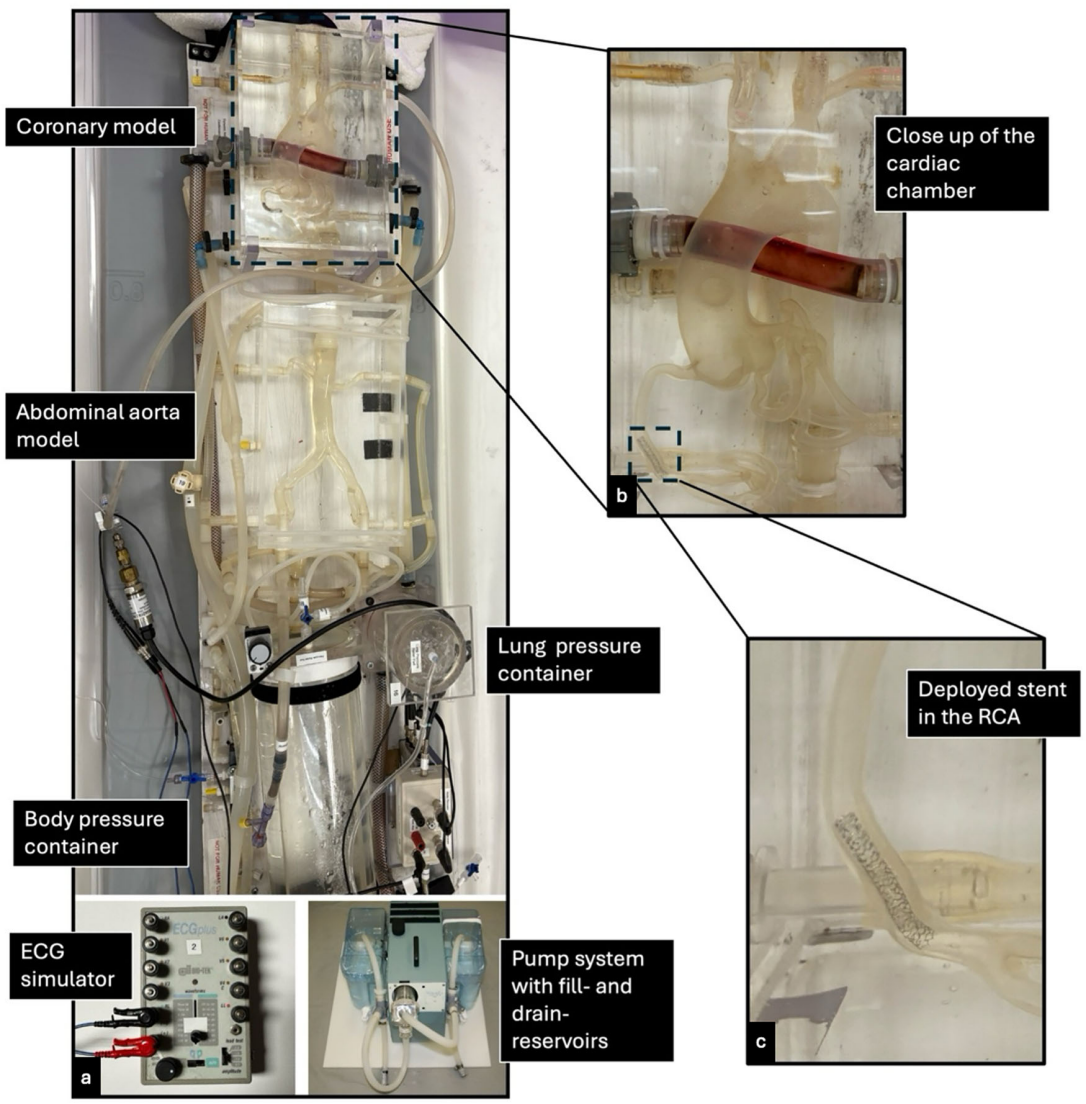
Overview of the phantom model used in this study (**a**), including the ECG simulator and pump. A zoomed portion of the coronary model (**b**) provides an anatomic overview of the aorta and its branches. At a further magnification, a deployed stent in the model of the right coronary artery (RCA) can be seen (**c**)

**Fig. 2:**
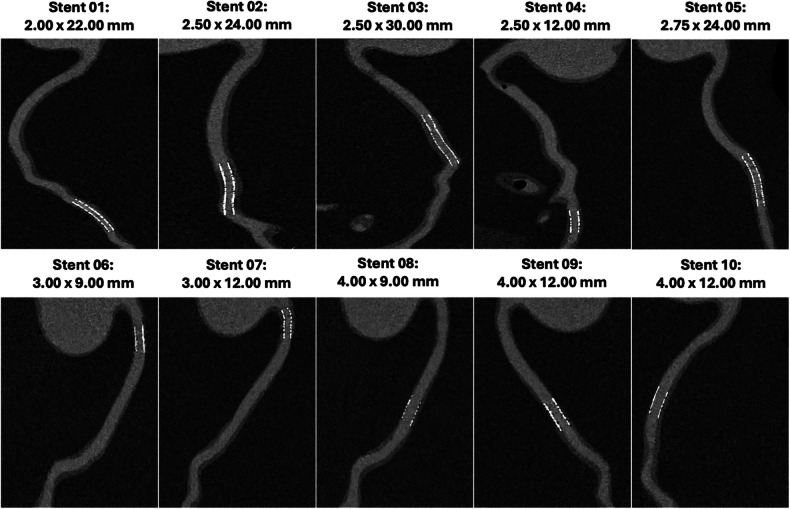
Curved multiplanar reconstructions (MPR) at ultrahigh-spatial resolution of all analyzed stents with diameters and lengths. The appearance of contrast medium surrounding Stents 02 and 03 results from the fixed vessel geometry of the phantom and curved MPR projection, not from true luminal pathology. Notably, even in small-sized stents, clear lumen visualization was achievable

A dynamic anthropomorphic circulatory phantom, modeling coronary arteries and vascular motion, was used in this study as previously described [18, 19]. The phantom consisted of plastic tubing linking a high-pressure and low-pressure chamber to replicate physiological hemodynamic conditions. Silicone vessels were used to simulate the coronary arteries and thoracic aorta (Model T-S-N-002; Elastrat), enclosed within an acrylic container filled with distilled water to emulate the background. The system was filled with 4 L of heated water maintained at 37 °C and circulated using a modified pulsatile pump to mimic cardiac contractions (BS4, Harvard Apparatus). An ECG simulator was connected to the pump and CT scanner for real-time synchronization with triggering at a single phase of 75% and at 300 ms for higher heart rates. Normal physiological conditions were modeled with heart rates set to 60, 80, and 100 beats per minute (bpm). Stroke volume was set to 90 mL to obtain a blood pressure of 120/80 mmHg. Stents were deployed in the right coronary artery following the manufacturer’s recommended specifications. For this, each stent was deployed under visual guidance by a board-certified radiologist with experience in interventional radiology (M.T.H.). Balloon inflation was carried out at the nominal pressure recommended by the manufacturer, with pressures ranging from 9 to 12 atmospheres, depending on the compliance chart. In total, 10 stents of different lengths, diameters, and materials were used. Table 1 outlines detailed stent characteristics. Figure 1 provides an overview of the experimental phantom setup, while Fig. 2 provides curved multiplanar reconstructions of all deployed stents.

**Table 1:** Stent characteristics

Stent ID	Manufacturer	Name	DES/BMS	Material composition	Diameter (mm)	Length (mm)	Nominal strut width (mm)*
01	Medtronic	Resolute Onyx	DES	Cobalt–platinum–iridium	2.00	22.00	0.081
02	Boston Scientific	Promus ELITE	DES	Platinum–Chromium	2.50	24.00	0.081
03	Medtronic	Resolute Integrity	DES	Cobalt–Chromium	2.50	30.00	0.091
04	Medtronic	Resolute Integrity	DES	Cobalt–Chromium	2.50	12.00	0.091
05	Boston Scientific	Synergy Monorail	DES	Platinum–Chromium	2.75	24.00	0.074
06	Medtronic	Resolute Integrity	DES	Cobalt–Chromium	3.00	9.00	0.091
07	Boston Scientific	Synergy Monorail	DES	Platinum–Chromium	3.00	12.00	0.074
08	Biotronik	Pro-Kinetic Energy	BMS	Cobalt–Chromium	4.00	9.00	0.060
09	Boston Scientific	Synergy Monorail	DES	Platinum–Chromium	4.00	12.00	0.081
10	Medtronic	Resolute Integrity	DES	Cobalt–Chromium	4.00	12.00	0.091

*DES* Drug-eluting stent, *BMS* Bare metal stent

* Derived from manufacturer’s specifications

### CT acquisition

All scans were performed on a clinical dual-source PCD-CT system (NAEOTOM Alpha, software version VB10, Siemens Healthineers). An anteroposterior scout view was initially acquired, followed by a retrospectively ECG-gated, spiral coronary CT angiography using a combined UHR and spectral mode. Scans were acquired at a peak tube voltage of 120 kVp. The tube rotation time was 250 ms, with a collimation of 96 × 0.2 mm. Automatic exposure control was applied (Care kV IQ level of 64). Iodinated contrast (Ultravist 370^®^, Iopromide, Bayer Healthcare) was administered via a dedicated injection port using a dual-syringe power injector (Medrad Stellant Flex, Bayer Healthcare). The contrast was delivered at a rate of 4 mL/s over 20 s, totaling 80 mL, followed by a 50 mL saline flush at the same rate. Scans were initiated using bolus tracking, with a region of interest (ROI) placed in the model aortic root. Scanning began once the measured attenuation exceeded a threshold of 110 HU, followed by a fixed post-trigger delay of 7 s.

### CT reconstruction

Polychromatic UHR images were reconstructed with a section thickness of 0.2 mm and an increment of 0.1 mm. Spectrally processed images were reconstructed at a section thickness of 0.4 mm with a 0.2 mm increment. Additionally, a downsampled polychromatic reconstruction was performed using a 0.6 mm section thickness, a 0.3 mm increment, and a softer convolution kernel (Bv44) to mimic the image impression of third-generation EID-CT systems [20]. To ensure isotropic voxel spacing, a field of view of 205 × 205 mm was used. Consequently, the matrix size for polychromatic UHR images was set at 1,024^2^ pixels, while spectrally processed images employed a 512^2^-pixel matrix. All images were reconstructed using a sharp vascular convolution kernel (Bv72) with iterative reconstruction (Quantum Iterative Reconstruction, level 3). Spectral post-processing included the following datasets: VMIs at 45, 55, 70, 85, and 100 keV; vessel lumen-preserving images (PURE Lumen, Siemens Healthineers); and IM images.

### Image analysis: general methods

Each reconstruction was independently assessed for both objective and subjective image criteria by two board-certified radiologists, with 7 years of experience each in cardiovascular imaging (M.T.H. and D.K.). Subjective image scores and multiplanar reformations were performed using Syngo.via (VB70A, CT Coronary Analysis, Siemens Healthineers). Objective image analysis, including measurement of stent lumen visibility, was conducted using Horos version 3.3.6 (The Horos Project, open-source, horosproject.org) and OsiriX (Pixmeo SARL), with OsiriX specifically used for its ability to copy and paste identical regions of interest across reconstructions, ensuring consistency in measurements. Both readers were blinded to reconstruction and stent characteristics during assessment.

### Objective image analysis

#### Lumen attenuation, noise, and contrast-to-noise ratio (CNR)

A total of five ROIs were manually drawn in the first reconstruction and copied to subsequent assessments to ensure maximum comparability. Two equal-sized circular ROIs were placed at least 1 mm before and after the stent inside the vessel lumen, while carefully avoiding the vessel wall. One oval ROI was placed along the stent lumen, carefully avoiding struts. Two larger circular ROIs were positioned parallel to the lumen ROIs, 10 mm from the vessel wall, to measure the attenuation and standard deviation (SD) of the background water. Lumen attenuation was the average attenuation of lumen ROIs. Noise corresponded to the average SD of the background ROIs. The CNR was calculated using the following equation:

$${{\rm{CNR}}}=\frac{{{{\rm{Attenuation}}}}_{{{\rm{Lumen}}}}\left[{{\rm{HU}}}\right]-{{{\rm{Attenuation}}}}_{{{\rm{Water}}}}[{{\rm{HU}}}]}{{{{\rm{SD}}}}_{{{\rm{Water}}}}[{{\rm{HU}}}]}$$

#### Measurable approximated stent lumen visibility

The maximum inner and outer diameters of the proximal and distal stent portions were manually measured on cross-axial reconstructions. Vessel diameters proximal and distal to the stent were measured semi-automatically on axial slices of curved planar reformations (Syngo.via, software version VB70A). Stent lumen visibility was calculated using the following equation:

$${\mathrm{Stent}}\,{\mathrm{lumen}}\,{\mathrm{visibility}}\,[ \% ]=\frac{{\mathrm{Interna}}\,{\mathrm{lstent}}\,{\mathrm{diameter}}\,[{\mathrm{mm}}]}{{\mathrm{Externa}}\,{\mathrm{lstent}}\,{\mathrm{diameter}}\,[{\mathrm{mm}}]}\times 100$$

#### Measurable strut width and overestimation factor

Measurable strut width was calculated by subtracting the measured inner diameter from the measured outer diameter. Consequently, the calculated strut-width was divided by the nominal values provided by the manufacturers to yield the stent-strut overestimation factor.

$${\mathrm{Overestimation}}\,{\mathrm{factor}}=\,\frac{{\mathrm{Measured}}\,{\mathrm{stent}}\,{\mathrm{strut}}\,{\mathrm{width}}\,[{\mathrm{mm}}]}{{\mathrm{Nominal}}\,{\mathrm{stent}}\,{\mathrm{strut}}\,{\mathrm{width}}\,[{\mathrm{mm}}]}$$

#### Kurtosis and full width at half maximum (FWHM)

Cross-sectional images of each stent were generated from the acquired dataset. Attenuation profiles perpendicular to the stent’s longitudinal axis, determined through multiplanar reconstructions, were then extracted, and an average attenuation profile was computed using specialized software (ImageJ2, version 2.13.0/1.54f, National Institutes of Health). From these profiles, the kurtosis, with the aim of assessing the peak sharpness and edge definition, and FWHM of the stent struts, to determine spatial resolution, was calculated.

### Subjective image analysis

All reconstructions were rated on axial and three-dimensional multiplanar projection views on a 4-point Likert scale as excellent (4 = excellent visualization of margins), good (3 = well-defined margins), poor (2 = blurred margins), and nondiagnostic (1 = margins not discernible). Four categories were graded: (i) stent lumen visibility as a surrogate marker for diagnostic confidence of stent patency; (ii) strut delineation as a marker of image sharpness; (iii) artifact severity, including motion artifacts and streak artifacts from partial volume averaging; and (iv) overall impression of image quality.

### Statistical analysis

All statistical analyses were performed using GraphPad Prism (version 10.4.1) and SPSS (version 30.0). To screen data for normal distribution, the Kolmogorov–Smirnov test was used. Quantitative variables were expressed as mean ± SD for normally distributed data or as median and interquartile range, when normality could not be assumed. Categorical variables were expressed as counts and percentages. For objective assessments, repeated-measures analysis of variance−ANOVA with Tukey *post hoc* test was used to correct for multiple comparisons. For subjective assessments within reconstructions, the Friedman test followed by the Dunn *post* *hoc* test was applied. Inter-reader agreement was assessed using weighted Cohen κ (quadratic weights) or the Friedman test (Wilcoxon signed rank test with Bonferroni correction was used as the *post hoc* test). Weighted κ was categorized as poor (< 0.01), slight agreement (0.01–0.20), fair agreement (0.21–0.40), moderate agreement (0.41–0.60), substantial agreement (0.61–0.80), or near perfect agreement (0.81–1.00). Results are presented with their corresponding 95% confidence intervals, if appropriate. A two-sided *p*-value of < 0.05 was considered statistically significant unless otherwise noted.

## Results

### Objective image quality

**Fig. 3:**
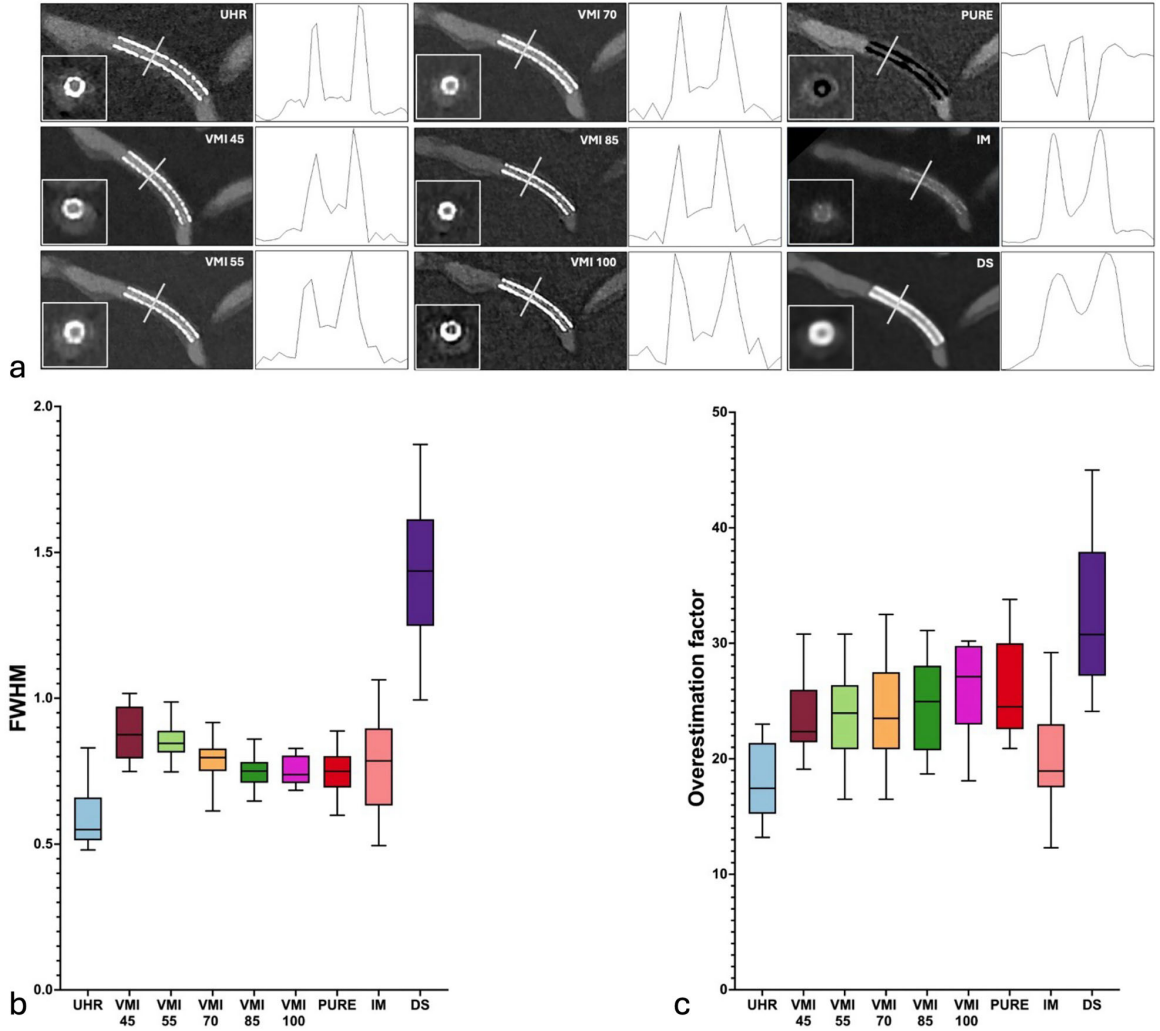
**a** Demonstrates multiplanar reconstructions of Stent 01 using all tested reconstruction techniques with corresponding cross-sectional images in the bottom left and FWHM curves to the right. Box and whisker plots for FWHM (**b**) and overestimation factor (**c**) demonstrate significantly sharper image characteristics for ultrahigh-resolution (UHR) mode compared to the VMI, pure lumen (PURE), and downsampled (DS) reconstructions, with the exception of IM

**Fig. 4:**
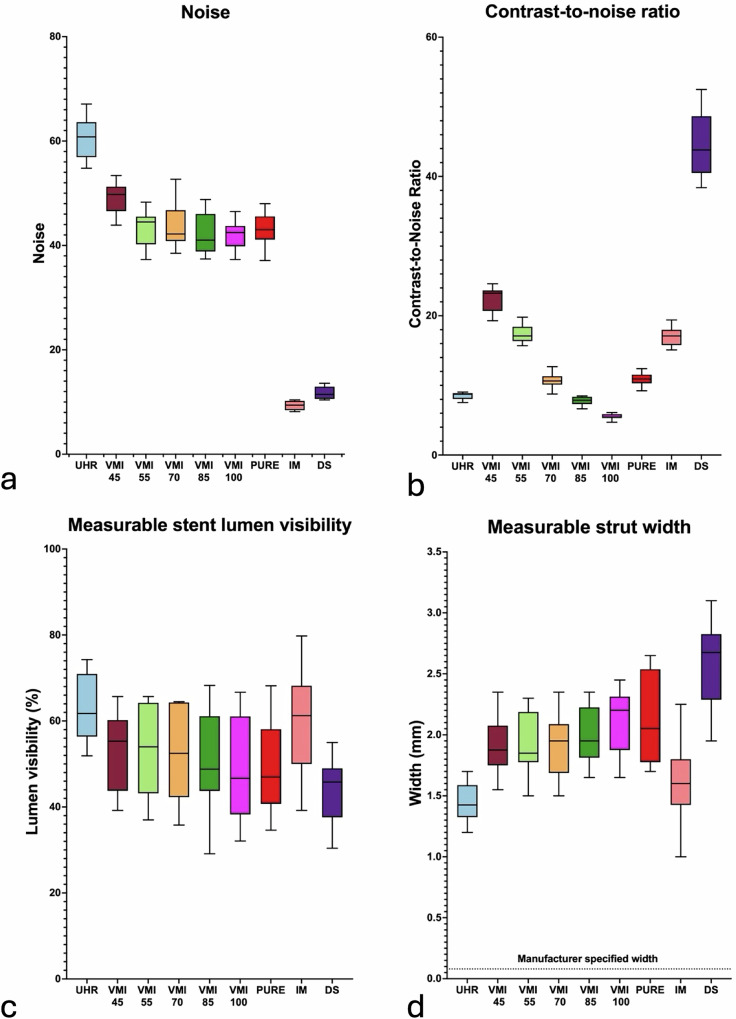
Box-and-whisker plots showing minimum and maximum values of all stents and reconstructions at 60 bpm for four objective image quality criteria: noise (**a**), CNR (**b**), stent lumen visibility (**c**), and measurable strut width (**d**). DS, Downsampled; IM, Iodine map; PURE, Lumen preserving spectral images; UHR, Ultrahigh-resolution; VMI, Virtual monoenergetic images

A comprehensive summary of all objective image quality results for various heart rates is listed in Table 2. Figure 3 provides a visual representation of stent 01 at 60 bpm using every applied reconstruction, with box and whisker plots for FWHM and the overestimation factor. Figure 4 summarizes box and whisker plots for noise, CNR, measurable stent lumen visibility, and measurable stent width for all reconstructions at a heart rate of 60 bpm. Intra-group comparisons for objective image criteria are provided in Tables S1, S2, and S3, for 60, 80, and 100 bpm, respectively. No significant differences were noted between objective image quality criteria at differing heart rates (all *p* > 0.050), except for kurtosis and FWHM (see below).

**Table 2:** Comparison of objective image quality metrics at varying heart rates using different parameters

	UHR	VMI 45	VMI 55	VMI 70	VMI 85	VMI 100	PURE	IM	DS	*p*-value
60 bpm										
Lumen attenuation (HU)	513 ± 19	1099 ± 46	751 ± 25	462 ± 20	325 ± 9	230 ± 14	466 ± 12	159 ± 7	521 ± 19	< 0.01
Noise (HU)	60.3 ± 4.0	49.1 ± 3.0	43.4 ± 3.5	43.6 ± 4.4	42.2 ± 4.0	41.9 ± 2.7	43.1 ± 3.3	9.3 ± 0.8	11.8 ± 1.1	< 0.01
CNR	8.5 ± 0.5	22.5 ± 1.7	17.4 ± 1.3	10.7 ± 1.1	7.8 ± 0.7	5.5 ± 0.4	10.9 ± 0.9	17.1 ± 1.4	44.6 ± 4.7	< 0.01
Stent lumen visibility (%)	62.6 ± 7.6	52.7 ± 9.1	52.7 ± 10.2	52.4 ± 10.5	50.6 ± 11.9	48.2 ± 11.8	49.1 ± 10.8	59.6 ± 11.9	43.5 ± 8.2	< 0.01
Measured strut width (mm)	1.45 ± 0.17	1.91 ± 0.24	1.92 ± 0.26	1.93 ± 0.28	2.01 ± 0.25	2.11 ± 0.27	2.21 ± 0.37	1.62 ± 0.34	2.57 ± 0.35	< 0.01
Overestimation factor	18.1 ± 3.4	23.7 ± 3.4	23.9 ± 4.1	24.1 ± 4.7	24.9 ± 4.1	26.2 ± 4.2	26.2 ± 4.6	20.2 ± 5.0	32.1 ± 6.5	< 0.01
FWHM	0.59 ± 0.11	0.88 ± 0.10	0.86 ± 0.07	0.79 ± 0.08	0.75 ± 0.06	0.75 ± 0.05	0.75 ± 0.08	0.77 ± 0.18	1.4 ± 0.09	< 0.01
Kurtosis	2.45 ± 0.47	1.51 ± 0.18	1.76 ± 0.38	1.96 ± 0.42	1.98 ± 0.45	2.08 ± 0.52	1.46 ± 0.17	1.68 ± 0.18	1.13 ± 0.36	< 0.01
80 bpm										
Lumen attenuation (HU)	519 ± 19	1087 ± 33	748 ± 23	468 ± 23	325 ± 15	238 ± 19	471 ± 21	150 ± 17	521 ± 26	< 0.01
Noise (HU)	59.2 ± 2.6	51.4 ± 4.4	44.7 ± 3.2	42.5 ± 2.3	41.9 ± 2.0	43.2 ± 2.0	43.4 ± 2.6	8.9 ± 0.6	15.7 ± 7.0	< 0.01
CNR	8.8 ± 0.4	21.3 ± 2.1	16.8 ± 1.2	11.0 ± 1.2	7.8 ± 0.3	5.5 ± 0.4	10.9 ± 0.5	16.9 ± 2.5	37.6 ± 11.2	< 0.01
Stent lumen visibility (%)	59.6 ± 9.4	55.2 ± 10.2	53.3 ± 9.4	50.2 ± 14.8	51.8 ± 10.9	48.9 ± 13.7	47.9 ± 10.5	59.0 ± 10.9	43.4 ± 7.7	< 0.01
Measured strut width (mm)	1.55 ± 0.12	1.80 ± 0.24	1.94 ± 0.28	2.04 ± 0.39	1.99 ± 0.25	2.12 ± 0.38	2.25 ± 0.37	1.63 ± 0.34	2.63 ± 0.34	< 0.01
Overestimation factor	19.3 ± 3.1	22.3 ± 3.2	24.1 ± 4.6	25.4 ± 5.7	24.9 ± 5.1	26.5 ± 6.5	28.1 ± 6.4	20.2 ± 4.6	32.9 ± 7.7	< 0.01
FWHM	0.71 ± 0.16	0.98 ± 0.18	0.99 ± 0.14	0.96 ± 0.31	0.88 ± 0.16	0.84 ± 0.19	0.75 ± 0.08	0.76 ± 0.16	1.3 ± 0.26	< 0.01
Kurtosis	2.28 ± 0.57	1.61 ± 0.49	1.60 ± 0.55	1.46 ± 0.52	1.85 ± 0.56	1.98 ± 0.74	1.43 ± 0.17	1.69 ± 0.16	1.02 ± 0.33	< 0.01
100 bpm										
Lumen attenuation (HU)	520 ± 20	1088 ± 41	737 ± 27	461 ± 22	319 ± 14	240 ± 21	461 ± 25	158 ± 6	519 ± 23	< 0.01
Noise (HU)	59.2 ± 3.1	49.1 ± 3.3	43.3 ± 3.4	41.6 ± 3.0	41.8 ± 3.3	41.7 ± 3.0	43.1 ± 3.4	9.2 ± 0.4	13.6 ± 5.9	< 0.01
CNR	8.8 ± 0.7	22.3 ± 1.8	17.1 ± 1.1	11.1 ± 0.7	7.7 ± 0.7	5.8 ± 0.6	10.8 ± 0.8	17.3 ± 1.2	41.6 ± 9.8	< 0.01
Stent lumen visibility (%)	59.2 ± 10.4	54.4 ± 10.6	53.7 ± 9.1	52.5 ± 13.1	50.5 ± 13.0	48.1 ± 14.2	46.1 ± 11.8	57.2 ± 10.5	45.6 ± 7.6	< 0.01
Measured strut width (mm)	1.60 ± 0.27	1.88 ± 0.22	1.95 ± 0.21	1.95 ± 0.30	2.06 ± 0.28	2.17 ± 0.34	2.28 ± 0.35	1.66 ± 0.32	2.54 ± 0.35	< 0.01
Overestimation factor	20.0 ± 4.8	23.4 ± 4.2	24.3 ± 4.4	24.2 ± 4.6	25.7 ± 5.1	27.1 ± 5.1	28.4 ± 5.7	20.5 ± 4.2	31.5 ± 5.6	< 0.01
FWHM	0.72 ± 0.18	1.04 ± 0.32	1.02 ± 0.17	0.91 ± 0.13	0.88 ± 0.14	0.85 ± 0.12	0.75 ± 0.08	0.75 ± 0.15	1.33 ± 0.18	< 0.01
Kurtosis	2.02 ± 0.76	1.28 ± 0.51	1.42 ± 0.35	1.52 ± 0.33	1.61 ± 0.40	1.95 ± 0.58	1.43 ± 0.18	1.69 ± 0.16	0.965 ± 0.46	< 0.01

IM values represent HU-equivalent IM outputs from the scanner; conversion to true iodine concentration requires multiplication by a constant factor (×0.03846 mg/mL), as provided by the vendor’s spectral basis decomposition calibration

*BMS* Bare metal stent, *bpm* Beats per min, *CNR* Contrast-to-noise ratio, *DES* Drug-eluting stent, *DS* Downsampled, *FWHM* Full width at half maximum, *IM* Iodine map, *PURE* Lumen preserving spectral images, *UHR* Ultrahigh-resolution, *VMI* Virtual monoenergetic images

* Derived from manufacturer’s specifications

#### Lumen attenuation, noise, and CNR

VMI reconstructions allowed for significantly higher attenuation values compared to UHR (60 bpm, 513 ± 19 HU), with an increase of up to 114% (*e.g*., VMI 45: 1,099 ± 46 HU at 60 bpm). CNR analysis showed the highest values for downsampled (44.6 ± 4.7), followed by VMI 45 (22.5 ± 1.7), VMI 55 (17.4 ± 1.3), and IM (17.1 ± 1.4), while UHR demonstrated intermediate values (8.5 ± 0.5) and VMI 100 the lowest (5.5 ± 0.4; all *p* < 0.001).

#### Measurable approximated stent lumen visibility

UHR performed significantly better than all other reconstructions regarding measurable stent lumen (UHR: 62.6 ± 7.6%, rest ranging from 48.2% to 52.7%, all *p* < 0.001), except IM (59.6 ± 11.9%, *p* = 0.839). IM similarly outperformed all other reconstructions except UHR (all *p* < 0.026). UHR at 80 bpm (59.2 ± 9.4%) and at 100 bpm (59.2 ± 10.4%) became comparable to VMI 45 (80 bpm: 55.2 ± 10.2%, *p* = 0.500; 100 bpm: 54.4 ± 10.6%, *p* = 0.244) and VMI 55 (80 bpm: 53.3 ± 9.4%, *p* = 0.101; 100 bpm: 53.7 ± 9.5%, *p* = 0.120).

#### Measured strut width and overestimation factor

Strut width on UHR images resulted in the closest measurements to the manufacturer-specified width (range, manufacturer: 0.060 to 0.091 mm; UHR: 1.20–1.70, *p* < 0.001). Downsampled reconstructions demonstrated an overestimation factor almost double that of UHR (18.1 ± 3.5 *versus* 32.1 ± 6.5, respectively; *p* < 0.001). IM was the only spectral-postprocessed reconstruction performing comparably to UHR (20.2 ± 5.0, pairwise *p* = 0.928).

#### Kurtosis and FWHM

UHR exhibited a significantly higher kurtosis and smaller FWHM than all other reconstructions (all *p* < 0.05), indicating the highest sharpness. Detailed results are summarized in Table 2.

### Subjective image quality

**Fig. 5:**
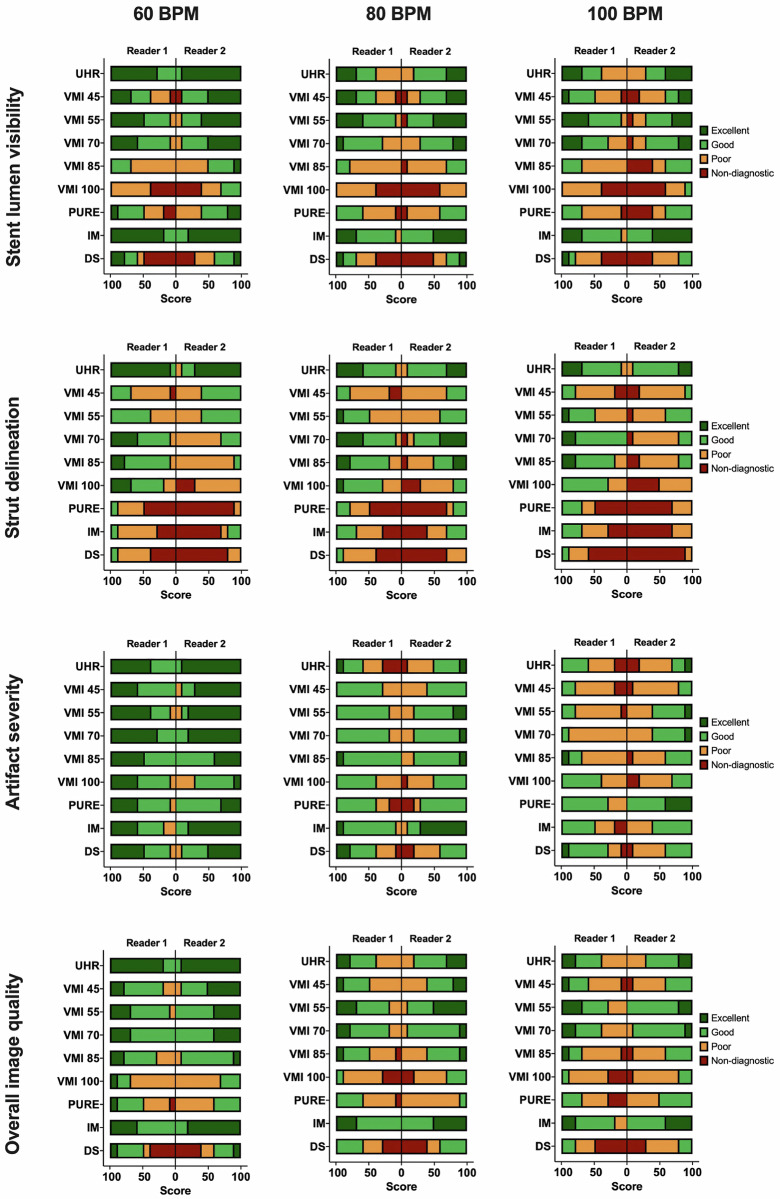
Stacked bar graphs showing the distribution of scores on a 4-point Likert scale for the subjective visual criteria stent lumen visibility, strut delineation, artifacts, and overall image quality for both readers. DS, Downsampled; IM, Iodine map; PURE, Lumen preserving spectral images; UHR, Ultrahigh-resolution; VMI, Virtual monoenergetic images

Overall, inter-reader agreement varied significantly, ranging from no agreement (Strut delineation for VMI 100 [κ 0.000]) to near-perfect agreement (*e.g*., downsampled overall image quality at 60 bpm [κ 0.914]). Figure 5 provides a visual representation of both readers’ subjective image quality scores for all stents at all heart rates. All heart rate-dependent inter-reader comparisons for 60, 80, and 100 bpm are summarized in Tables S4, S5, and S6, respectively. Detailed results comparing all subjective image criteria between all heart rates for each reader are provided in Table S7.

#### Subjective stent lumen visibility

For both readers, UHR at 60 bpm achieved the highest diagnostic confidence of stent patency (Reader 1: 4 [IQR 3.3–4], Reader 2: 4 [4]) followed by IM (Reader 1: 4 [IQR 3.8–4], Reader 2: 4 [3.8–4]). VMI 55 achieved the best score out of all VMIs (Reader 1: 3.5 [IQR 3–4], Reader 2: 4 [3, 4]). UHR and IM were the only two reconstructions with significant differences in stent lumen visibility between all heart rates for both readers (all *p* < 0.050).

#### Stent-strut delineation as a marker of image sharpness

Similarly, UHR achieved the highest scores for both readers regarding image sharpness based on strut delineation (Reader 1: 4 [IQR 4–4], Reader 2: 4 [IQR 3–4]) with VMI 55 achieving the second-best results (Reader 1: 3.0 [IQR 2–3], Reader 2: 3 [IQR 2–3]). Neither Reader 1 nor 2 rated UHR significantly better than VMI 55, although UHR performed markedly better than other VMIs, PURE, IM, and downsampled (all *p* > 0.050).

#### Artifact severity, including motion artifacts and streak artifacts from partial volume averaging

Artifact severity did not differ significantly for any groups at 60 bpm, though Reader 1 reported fewer artifacts on UHR compared to VMI 100 (*p* = 0.031). At heart rates of 80 and 100 bpm, UHR scored consistently less for both readers compared to VMIs, although without a significant difference (all *p* > 0.050).

#### Overall impression of image quality

UHR achieved the highest scores for both readers (Reader 1: 4 [IQR 3.8–4]; Reader 2: 4 [IQR 4–4]) at 60 bpm, although these scores were only significantly better than VMI 85 (Reader 1: 3 [IQR 2–3.3], *p* = 0.045) and VMI 100 (Reader 1: 2 [IQR 2–3], *p* < 0.001; Reader 2: 2 [IQR 2–3], *p* < 0.001).

## Discussion

We sought to systematically compare coronary stent visualization in a dynamic, anthropomorphic coronary vessel phantom using a novel PCD-CT scanning method allowing for the simultaneous UHR and spectral acquisition. Our main findings are as follows:

i. Polychromatic UHR images consistently demonstrated the highest in stent lumen visibility across all heart rates, significantly outperforming downsampled and all VMIs, with a maximum visibility of 62.6 ± 7.6% at 60 bpm (*p* < 0.01), as well as providing, together with IM, the lowest overestimation factor of stent struts;
ii. VMIs at lower keV increased the CNR (VMI 45 ranging from 21 ± 2.1 to 22.5 ± 1.7) but had lower sharpness;
iii. UHR images were more susceptible to image quality degradation caused by motion artifacts at heart rates of 80 and 100 bpm; here, IM and VMIs showed more consistent and promising performance.

Collectively, these findings therefore demonstrate the incremental value of combining UHR and spectral PCD-CT.

The recent introduction of a clinically approved PCD-CT system in daily clinical routine has led to advancements in intraluminal stent imaging due to improved spatial resolution and reduced metal artifacts, as reported in previous studies [21–24]. Our findings are in line with other contemporary studies, suggesting that a decrease in partial volume averaging artifacts translates into an increase in in-stent lumen visibility [12, 25]. Similarly, we noted superior image sharpness in the UHR images, as demonstrated by the more accurate strut width measurements and consequently the lowest strut overestimation factor out of all reconstructions. Comparable results were also observed by other investigations, reporting increased image sharpness of PCD-CT leading to higher in-stent lumen visibility [21, 24, 25]. However, a thinner slice selection and a smaller focal point both contribute to the scanner’s ability to examine smaller structures, beyond stent assessment, as recent studies have shown potential in interstitial lung imaging or temporal bone assessment. This is corroborated by the fact that 0.2 mm slice thickness UHR images consistently scored higher than 0.4 mm slice thickness spectral images regarding image sharpness in our study, in the context of stent visualization. For spectral processed images, VMI 55 consistently demonstrated better objective and subjective image quality scores than other VMIs, confirming results reported by Elias Michael et al [26]: they found that VMI 55 outperformed VMIs at 65, 75, 90, and 110 keV for coronary imaging. Similarly, Higashigaito et al [27] reported VMIs at 55 keV to provide improved vascular assessment for the aorta. Gnasso et al [28] reported improved agreement with MRI for quantification of extracellular volume using lower energy VMIs. We attribute these findings to a preference of readers for a higher CNR when assessing smaller objects of interest, unless noise increases in very low energy VMIs [29].

Whilst the results presented in this study demonstrate overall agreement with previously published works, they differ in two key areas: firstly, a dynamic, moving phantom was employed to explore the effect of motion, and secondly, UHR and spectral data were acquired simultaneously. At first glance, an in-stent lumen visibility of 62.6% for UHR PCD-CT might not seem like a generational leap compared to the 50–59% reported by Maintz et al in 2008 for EID-CTs [30]. However, we attribute this observation to differences in phantom model design. The ECG-triggered pulsatile motion in our dynamic model introduces motion-related blurring, even at 60 bpm, which is a factor that is not present in stationary models. Importantly, for polychromatic UHR, susceptibility to motion artifacts was markedly elevated compared to other reconstructions. These results are confirmatory to prior investigations, as a recent study reported degradation in image quality at simulated coronary CT angiography acquisition with reduced temporal resolution in 30 subjects using UHR [31]. The increased risk of image quality degradation when UHR PCD-CT is performed is not unique to coronary imaging: A recent phantom study investigated pancreatic motion and observed image quality deterioration when acquisitions with the highest spatial resolution were performed [32]. All of these results point to the need for further improvement of UHR-specific motion correction algorithms and for faster data transfer, as the mentioned collimation is not a technical limitation of the PCD technology. Intriguingly, IM offered fewer artifacts and, next to polychromatic UHR images, the highest sharpness, indicated by the lowest FWHM and overestimation factor. IM has traditionally been utilized in cardiac imaging for myocardial tissue characterization, particularly for late iodine enhancement and extracellular volume quantification [33–35].

However, it remains to be investigated whether IMs can further be used for vascular, or stent lumen assessment, as the ongoing advancement in PCD-CT technology with improved spatial resolution may contribute to the use of IMs as a reliable spectral alternative in vascular assessment. For PCD-CT, IM has been reported to provide excellent performance in oncological imaging, notably in breast cancer patients [36, 37]. Notably, vessel-lumen preserving images performed poorly for in-stent lumen visualization. This is not a surprising result, as this algorithm is based on a multi-energy iodine and calcium separation and was not developed for metallic stent materials such as cadmium, or platinum [38]. Previous investigation of this algorithm has shown improved stenosis accuracy compared to standard resolution images *in vitro* and *in vivo* [39, 40]. Of note, the sharp Bv72 kernel was used for both UHR and spectral reconstructions, as it is clinically recommended for stent imaging [41]. This consistent application ensured that differences in image quality could be primarily attributed to acquisition geometry and voxel size, rather than post-processing variability. However, this might be disadvantageous in spectral images with lower intrinsic sampling density, as the sharp kernel might amplify noise without providing equivalent gains in spatial resolution. Another consideration before clinical implementation is, that the combined UHR and spectral protocol uses a reduced 96 × 0.2 mm collimation, which may lead to increased acquisition times compared to conventional modes. This may require careful planning regarding contrast media volume, timing, and breath-hold training. However, a faster pitch setting could, in theory, offset prolonged acquisition time and mitigate motion-related challenges in clinical workflows.

The following limitations of our study merit consideration. First, the use of an *ex vivo* dynamic anthropomorphic phantom, while enabling controlled and reproducible conditions, cannot fully mimic the complexities of human anatomy and physiology; therefore, the clinical applicability of our results requires separate evaluation. Second, the study lacks *in vivo* validation. Third, the evaluation of ten coronary stents with specific materials and dimensions may not fully represent the diversity of stent designs in clinical practice, reducing the generalizability of the findings. Fourth, all used stents were patent, and no model of in-stent stenosis was available. Fifth, while our phantom setup allows for a more physiologic contrast injection and a realistic anatomical structure, its motion characteristics remain distinct from those of dedicated motion phantoms. Lastly, the use of a uniformly sharp kernel across all reconstructions may have disproportionately increased noise in spectral modes. Future *ex vivo* studies should investigate the impact of K-edge imaging with multiple energy binning and evaluate the performance of novel contrast agents containing high-Z materials, which may offer advantages in cardiovascular imaging [42].

To conclude, our study demonstrates the advantages of combining UHR and spectral imaging in PCD-CT for coronary stent visualization, enhancing both objective and subjective image quality. While UHR imaging excelled in stent lumen visibility and image sharpness, spectral post-processed images provided complementary value in increasing CNR or in reducing artifacts at higher heart rates.

## Supplementary information

**Additional file 1: Table S1.** Summary of Tukey’s multiple comparison test after repeated-measures one-way ANOVA of objective image quality markers at 60 beats per minute. **Table S2.** Summary of Tukey’s multiple comparison test after repeated-measures one-way ANOVA of objective image quality markers at 80 beats per minute. **Table S3.** Summary of Tukey’s multiple comparison test after repeated-measures one-way ANOVA of objective image quality markers at 100 beats per minute. **Table S4.** Subjective image quality rating for all stents and reconstructions at 60 beats per min. **Table S5.** Subjective image quality rating for all stents and reconstructions at 80 beats per min. **Table S6.** Subjective image quality rating for all stents and reconstructions at 100 beats per min. **Table S7.** Results of Friedman’s test followed by Wilcoxon signed rank test for each reader between all heart rates.

## Data Availability

The datasets generated and/or analyzed during the current study are available from the corresponding author on reasonable request.

## Abbreviations

bpm: Beats per minute
CNR: Contrast-to-noise ratio
CT: Computed tomography
ECG: Electrocardiogram
EID: Energy-integrating detector
FWHM: Full width at half maximum
IM: Iodine map
PCD: Photon-counting detector
ROI: Region of interest
SD: Standard deviation
UHR: Ultrahigh-resolution
VMI: Virtual monoenergetic images
