# Supplementary material for: Spectral ultrahigh-resolution photon-counting CT for coronary stent imaging: evaluation in a dynamic phantom
Source: Eur Radiol Exp. 2025 Dec 2;9:115. doi: 10.1186/s41747-025-00654-2 (PMC12672982; doi:10.1186/s41747-025-00654-2)
Supplement: Supplementary file 1 — Additional file 1: Table S1. Summary of Tukey’s multiple comparison test after repeated-measures one-way ANOVA of objective image quality markers at 60 beats per minute. Table S2. Summary of Tukey’s multiple comparison test after repeated-measures one-way ANOVA of objective image quality markers at 80 beats per minute. Table S3. Summary of Tukey’s multiple comparison test after repeated-measures one-way ANOVA of objective image quality markers at 100 beats per minute. Table S4. Subjective image quality rating for all stents and reconstructions at 60 beats per min. Table S5. Subjective image quality rating for all stents and reconstructions at 80 beats per min. Table S6. Subjective image quality rating for all stents and reconstructions at 100 beats per min. Table S7. Results of Friedman’s test followed by Wilcoxon signed rank test for each reader between all heart rates. [file 41747_2025_654_MOESM1_ESM.pdf]

Spectral ultrahigh-resolution photon-counting CT for coronary stent imaging: evaluation in a dynamic phantom

ELECTRONIC SUPPLEMENTARY MATERIAL

Table S1: Summary of Tukey's multiple comparison test after repeated-measures one-way ANOVA of objective image quality markers at 60 beats per minute.

Reconstruction	Lumen attenuation	Noise	CNR	Lumen visibility	Strut width	Overestimation factor	FWHM	Kurtosis
UHR vs. VMI 45	<0.001	<0.001	<0.001	<0.001	0.003	0.002	<0.001	<0.001
UHR vs. VMI 55	<0.001	<0.001	<0.001	<0.001	0.001	0.001	<0.001	<0.001
UHR vs. VMI 70	<0.001	<0.001	0.001	<0.001	0.001	0.001	0.008	0.034
UHR vs. VMI 85	<0.001	<0.001	0.230	<0.001	<0.001	<0.001	0.053	0.048
UHR vs. VMI 100	<0.001	<0.001	<0.001	<0.001	<0.001	<0.001	0.060	0.262
UHR vs. PURE	<0.001	<0.001	<0.001	<0.001	0.001	<0.001	0.065	<0.001
UHR vs. IM	<0.001	<0.001	<0.001	0.839	0.928	0.809	0.018	<0.001
UHR vs. DS	0.967	<0.001	<0.001	<0.001	<0.001	<0.001	<0.001	<0.001
VMI 45 vs. VMI 55	<0.001	0.002	0.001	>0.999	>0.999	>0.999	>0.999	0.776
VMI 45 vs. VMI 70	<0.001	0.003	<0.001	>0.999	>0.999	>0.999	0.640	0.083
VMI 45 vs. VMI 85	<0.001	<0.001	<0.001	0.977	0.973	0.986	0.234	0.060
VMI 45 vs. VMI 100	<0.001	<0.001	<0.001	0.386	0.421	0.589	0.215	0.007
VMI 45 vs. PURE	<0.001	0.001	<0.001	0.684	0.239	0.620	0.201	>0.999
VMI 45 vs. IM	<0.001	<0.001	<0.001	0.025	0.494	0.191	0.449	0.970
VMI 45 vs. DS	<0.0001	<0.001	<0.001	0.001	0.001	<0.001	<0.001	0.202
VMI 55 vs. VMI 70	<0.001	>0.999	<0.001	>0.999	>0.999	>0.999	0.903	0.910
VMI 55 vs. VMI 85	<0.001	0.992	<0.001	0.975	0.950	0.996	0.518	0.859
VMI 55 vs. VMI 100	<0.001	0.971	<0.001	0.377	0.054	0.704	0.490	0.403
VMI 55 vs. PURE	<0.001	>0.999	<0.001	0.674	0.413	0.732	0.468	0.522
VMI 55 vs. IM	<0.001	<0.001	>0.999	0.026	0.539	0.131	0.768	0.999
VMI 55 vs. DS	<0.001	<0.001	<0.001	0.001	0.002	<0.001	<0.001	0.002
VMI 70 vs. VMI 85	<0.001	0.982	<0.001	0.992	0.982	0.999	0.999	>0.999
VMI 70 vs. VMI 100	<0.001	0.945	<0.001	0.492	0.195	0.785	0.998	0.994
VMI 70 vs. PURE	0.999	>0.999	0.999	0.784	0.279	0.810	0.997	0.029
VMI 70 vs. IM	<0.001	<0.001	<0.001	0.016	0.533	0.095	>0.999	0.626
VMI 70 vs. DS	<0.001	<0.001	<0.001	0.001	0.004	<0.001	<0.001	<0.001
VMI 85 vs. VMI 100	<0.001	>0.999	<0.001	0.958	0.643	0.988	>0.999	0.998
VMI 85 vs. PURE	<0.001	0.999	<0.001	0.998	0.894	0.992	>0.999	0.020
VMI 85 vs. IM	<0.001	<0.001	<0.001	0.001	0.351	0.015	>0.999	0.539
VMI 85 vs. DS	<0.001	<0.001	<0.001	0.019	0.017	<0.001	<0.001	<0.001
VMI 100 vs. PURE	<0.001	0.991	<0.001	>0.999	>0.999	>0.999	>0.999	0.002
VMI 100 vs. IM	<0.001	<0.001	<0.001	<0.001	0.099	0.001	>0.999	0.148
VMI 100 vs. DS	<0.001	<0.001	<0.001	0.319	0.068	0.001	<0.001	<0.001
PURE vs. IM	<0.001	<0.001	<0.001	<0.001	0.271	0.001	>0.999	0.847
PURE vs. DS	<0.001	<0.001	<0.001	0.129	0.061	0.001	<0.001	0.409
IM vs. DS	<0.001	0.651	<0.001	<0.001	0.010	<0.001	<0.001	0.011

CNR Contrast-to-noise ratio, *DS* Downsampled, *FWHM* Full width at half maximum, *IM* Iodine map, *PURE* Lumen preserving spectral images, *UHR* Ultrahigh-resolution, *VMI* Virtual monoenergetic images.

Table S2 Summary of Tukey's multiple comparison test after repeated-measures one-way ANOVA of objective image quality markers at 80 beats per minute.

Reconstruction	Lumen attenuation	Noise	CNR	Lumen visibility	Strut width	Overestimation factor	FWHM	Kurtosis
UHR vs. VMI 45	<0.001	<0.001	<0.001	0.500	0.055	0.563	0.003	0.043
UHR vs. VMI 55	<0.001	<0.001	<0.001	0.101	0.006	0.056	0.002	0.036
UHR vs. VMI 70	<0.001	<0.001	<0.001	0.001	0.013	0.004	0.009	0.004
UHR vs. VMI 85	<0.001	<0.001	0.002	0.014	<0.001	0.012	0.224	0.468
UHR vs. VMI 100	<0.001	<0.001	<0.001	<0.001	0.004	<0.001	0.596	0.875
UHR vs. PURE	<0.001	<0.001	<0.001	<0.001	<0.001	<0.001	>0.999	0.003
UHR vs. IM	<0.001	<0.001	<0.001	>0.999	0.999	>0.999	0.998	0.115
UHR vs. DS	>0.999	<0.001	<0.001	<0.001	<0.001	<0.001	<0.001	<0.001
VMI 45 vs. VMI 55	<0.001	<0.001	<0.001	0.995	0.257	0.959	>0.999	>0.999
VMI 45 vs. VMI 70	<0.001	<0.001	<0.001	0.355	0.343	0.515	>0.999	0.998
VMI 45 vs. VMI 85	<0.001	<0.001	<0.001	0.818	0.343	0.734	0.836	0.967
VMI 45 vs. VMI 100	<0.001	<0.001	<0.001	0.101	0.135	0.143	0.444	0.678
VMI 45 vs. PURE	<0.001	<0.001	<0.001	0.030	0.042	0.009	0.020	0.992
VMI 45 vs. IM	<0.001	<0.001	0.004	0.667	0.888	0.895	0.031	>0.999
VMI 45 vs. DS	<0.001	<0.001	0.019	<0.001	0.002	<0.001	<0.001	0.112
VMI 55 vs. VMI 70	<0.001	0.815	<0.001	0.873	0.974	0.994	>0.999	0.999
VMI 55 vs. VMI 85	<0.001	0.582	<0.001	0.998	0.987	>0.999	0.735	0.954
VMI 55 vs. VMI 100	<0.001	0.979	<0.001	0.500	0.544	0.806	0.333	0.633
VMI 55 vs. PURE	<0.001	0.992	<0.001	0.231	0.094	0.198	0.011	0.995
VMI 55 vs. IM	<0.001	<0.001	>0.999	0.177	0.502	0.217	0.019	>0.999
VMI 55 vs. DS	<0.001	<0.001	0.004	<0.001	0.003	<0.001	<0.001	0.131
VMI 70 vs. VMI 85	<0.001	>0.999	<0.001	0.998	0.999	>0.999	0.948	0.618
VMI 70 vs. VMI 100	<0.001	>0.999	<0.001	>0.999	0.984	0.998	0.648	0.218
VMI 70 vs. PURE	>0.999	>0.999	0.998	0.974	0.544	0.718	0.047	>0.999
VMI 70 vs. IM	<0.001	<0.001	<0.001	0.003	0.360	0.025	0.071	0.963
VMI 70 vs. DS	<0.001	<0.001	<0.001	0.052	0.086	<0.001	<0.001	0.474
VMI 85 vs. VMI 100	<0.001	0.992	<0.001	0.912	0.544	0.979	>0.999	0.999
VMI 85 vs. PURE	<0.001	0.980	<0.001	0.670	0.124	0.497	0.551	0.520
VMI 85 vs. IM	<0.001	<0.001	<0.001	0.029	0.436	0.062	0.660	0.998
VMI 85 vs. DS	<0.001	<0.001	<0.001	0.006	0.009	<0.001	<0.001	0.004
VMI 100 vs. PURE	<0.001	>0.999	<0.001	>0.999	0.880	0.982	0.905	0.161
VMI 100 vs. IM	<0.001	<0.001	<0.001	<0.001	0.210	0.003	0.952	0.889
VMI 100 vs. DS	<0.001	<0.001	<0.001	0.218	0.145	0.003	<0.001	<0.001
PURE vs. IM	<0.001	<0.001	<0.001	<0.001	0.083	<0.001	>0.999	0.928
PURE vs. DS	<0.001	<0.001	<0.001	0.482	0.239	0.057	<0.001	0.571
IM vs. DS	<0.001	<0.001	0.007	<0.001	0.005	<0.001	<0.001	0.042

CNR Contrast-to-noise ratio, *DS* Downsampled, *FWHM* Full width at half maximum, *IM* Iodine map, *PURE* Lumen preserving spectral images, *UHR* Ultrahigh-resolution, *VMI* Virtual monoenergetic images.

Table S3 Summary of Tukey's multiple comparison test after repeated-measures one-way ANOVA of objective image quality markers at 100 beats per minute.

Reconstruction	Lumen attenuation	Noise	CNR	Lumen visibility	Strut width	Overestimation factor	FWHM	Kurtosis
UHR vs. VMI 45	<0.001	<0.001	<0.001	0.244	0.007	0.202	<0.001	0.002
UHR vs. VMI 55	<0.001	<0.001	<0.001	0.120	0.003	0.045	<0.001	0.024
UHR vs. VMI 70	<0.001	<0.001	<0.001	0.022	0.093	0.053	0.077	0.113
UHR vs. VMI 85	<0.001	<0.001	0.003	<0.001	<0.001	0.001	0.265	0.321
UHR vs. VMI 100	<0.001	<0.001	<0.001	<0.001	<0.001	<0.001	0.500	>0.999
UHR vs. PURE	<0.001	<0.001	<0.001	<0.001	<0.001	<0.001	>0.999	0.029
UHR vs. IM	<0.001	<0.001	<0.001	0.982	>0.999	>0.999	>0.999	0.597
UHR vs. DS	>0.999	<0.001	<0.001	<0.001	<0.001	<0.001	<0.001	<0.001
VMI 45 vs. VMI 55	<0.001	0.005	<0.001	>0.999	0.888	>0.999	>0.999	0.996
VMI 45 vs. VMI 70	<0.001	<0.001	<0.001	0.986	0.998	>0.999	0.531	0.888
VMI 45 vs. VMI 85	<0.001	<0.001	<0.001	0.533	0.222	0.729	0.211	0.597
VMI 45 vs. VMI 100	<0.001	<0.001	<0.001	0.044	0.036	0.142	0.087	0.007
VMI 45 vs. PURE	<0.001	0.003	<0.001	0.002	0.030	0.010	<0.001	0.993
VMI 45 vs. IM	<0.001	<0.001	<0.001	0.857	0.582	0.412	<0.001	0.321
VMI 45 vs. DS	<0.001	<0.001	0.004	<0.001	0.003	<0.001	<0.001	0.684
VMI 55 vs. VMI 70	<0.001	0.973	<0.001	>0.999	>0.999	>0.999	0.734	>0.999
VMI 55 vs. VMI 85	<0.001	0.983	<0.001	0.753	0.832	0.974	0.369	0.972
VMI 55 vs. VMI 100	<0.001	0.980	<0.001	0.103	0.299	0.455	0.176	0.072
VMI 55 vs. PURE	<0.001	>0.999	<0.001	0.005	0.014	0.060	0.001	>0.999
VMI 55 vs. IM	<0.001	<0.001	>0.999	0.665	0.398	0.122	0.002	0.833
VMI 55 vs. DS	<0.001	<0.001	<0.001	0.002	0.005	<0.001	<0.001	0.197
VMI 70 vs. VMI 85	<0.001	>0.999	<0.001	0.981	0.916	0.964	>0.999	>0.999
VMI 70 vs. VMI 100	<0.001	>0.999	<0.001	0.379	0.397	0.417	0.989	0.266
VMI 70 vs. PURE	>0.999	0.988	0.961	0.039	0.269	0.051	0.191	>0.999
VMI 70 vs. IM	<0.001	<0.001	<0.001	0.262	0.147	0.140	0.227	0.989
VMI 70 vs. DS	<0.001	<0.001	<0.001	0.017	0.120	<0.001	<0.001	0.048
VMI 85 vs. VMI 100	<0.001	>0.999	<0.001	0.948	0.280	0.980	>0.999	0.580
VMI 85 vs. PURE	<0.001	0.993	<0.001	0.382	0.539	0.525	0.499	0.981
VMI 85 vs. IM	<0.001	<0.001	<0.001	0.021	0.247	0.006	0.557	>0.999
VMI 85 vs. DS	<0.001	<0.001	<0.001	0.230	0.115	0.002	<0.001	0.011
VMI 100 vs. PURE	<0.001	0.992	<0.001	0.981	0.962	0.986	0.757	0.085
VMI 100 vs. IM	<0.001	<0.001	<0.001	<0.001	0.089	<0.001	0.806	0.842
VMI 100 vs. DS	<0.001	<0.001	<0.001	0.923	0.411	0.040	<0.001	<0.001
PURE vs. IM	<0.001	<0.001	<0.001	<0.001	0.034	<0.001	>0.999	0.865
PURE vs. DS	<0.001	<0.001	<0.001	>0.999	0.707	0.362	<0.001	0.171
IM vs. DS	<0.001	0.074	<0.001	<0.001	0.008	<0.001	<0.001	0.003

CNR Contrast-to-noise ratio, *DS* Downsampled, *FWHM* Full width at half maximum, *IM* Iodine map, *PURE* Lumen preserving spectral images, *UHR* Ultrahigh-resolution, *VMI* Virtual monoenergetic images.

Table S4 Subjective image quality rating for all stents and reconstructions at 60 beats per min

	Parameter	Reader 1	Reader 2	Weighted κ	*p*-value
UHR	Stent lumen visibility	4.0 [3.3 - 4.0]	4.0 [4.0 - 4.0]	0.412	0.107
	Strut delineation	4.0 [4.0 - 4.0]	4.0 [3.3 - 4.0]	-0.136	0.490
	Artifact severity	4.0 [3.0 - 4.0]	4.0 [4.0 - 4.0]	0.286	0.197
	Overall image quality	4.0 [4.0 - 4.0]	4.0 [4.0 - 4.0]	0.615	**0.035**
VMI 45	Stent lumen visibility	3.0 [2.0 - 3.8]	3.5 [3.0 - 4.0]	0.545	**0.004**
	Strut delineation	2.0 [2.0 - 2.8]	3.0 [2.0 - 3.0]	0.063	0.778
	Artifact severity	3.0 [3.0 - 4.0]	4.0 [3.3 - 4.0]	-0.250	0.260
	Overall image quality	3.0 [3.0 - 3.0]	3.5 [3.0 - 4.0]	0.211	0.295
VMI 55	Stent lumen visibility	3.5 [3.0 - 4.0]	4.0 [3.0 - 4.0]	0.853	**0.001**
	Strut delineation	3.0 [2.0 - 3.0]	3.0 [2.0 - 3.0]	0.167	0.598
	Artifact severity	4.0 [3.0 - 4.0]	4.0 [4.0 - 4.0]	-0.290	0.230
	Overall image quality	3.0 [3.0 - 3.8]	3.0 [3.0 - 4.0]	0.286	0.260
VMI 70	Stent lumen visibility	3.0 [3.0 - 4.0]	3.5 [3.0 - 4.0]	0.559	**0.025**
	Strut delineation	3.0 [3.0 - 4.0]	2.0 [2.0 - 2.8]	0.057	0.490
	Artifact severity	4.0 [3.3 - 4.0]	4.0 [4.0 - 4.0]	-0.316	0.301
	Overall image quality	3.0 [3.0 - 3.8]	3.0 [3.0 - 4.0]	0.348	0.260
VMI 85	Stent lumen visibility	2.0 [2.0 - 2.8]	2.5 [2.0 - 3.0]	0.500	**0.038**
	Strut delineation	3.0 [3.0 - 3.0]	2.0 [2.0 - 2.0]	0.020	0.725
	Artifact severity	3.5 [3.0 - 4.0]	3.0 [3.0 - 4.0]	-0.400	0.197
	Overall image quality	3.0 [2.3 - 3.0]	3.0 [3.0 - 3.0]	0.167	0.395
VMI 100	Stent lumen visibility	2.0 [1.0 - 2.0]	2.0 [1.0 - 2.8]	0.103	0.598
	Strut delineation	3.0 [3.0 - 3.8]	2.0 [1.3 - 2.0]	0.000	>0.999
	Artifact severity	3.0 [3.0 - 4.0]	3.0 [2.3 - 3.0]	-0.184	0.285
	Overall image quality	2.0 [2.0 - 2.8]	2.0 [2.0 - 2.8]	0.808	**0.002**
PURE	Stent lumen visibility	2.5 [2.0 - 3.0]	3.0 [2.0 - 3.0]	0.167	0.378
	Strut delineation	1.5 [1.0 - 2.0]	1.0 [1.0 - 1.0]	0.167	0.292
	Artifact severity	3.0 [3.0 - 4.0]	3.0 [3.0 - 3.8]	-0.429	0.091
	Overall image quality	2.5 [2.0 - 3.0]	2.0 [2.0 - 3.0]	0.286	0.197
IM	Stent lumen visibility	4.0 [4.0 - 4.0]	4.0 [4.0 - 4.0]	-0.250	0.429
	Strut delineation	2.0 [1.3 - 2.0]	1.0 [1.0 - 1.8]	0.167	0.392
	Artifact severity	3.0 [3.0 - 4.0]	4.0 [4.0 - 4.0]	-0.053	0.747

	Overall image quality	3.0 [3.0 - 4.0]	4.0 [4.0 - 4.0]	-0.071	0.747
DS	Stent lumen visibility	1.5 [1.0 - 3.0]	2.0 [1.3 - 3.0]	0.597	**0.008**
	Strut delineation	2.0 [1.0 - 2.0]	1.0 [1.0 - 1.0]	0.242	0.197
	Artifact severity	3.5 [3.0 - 4.0]	3.5 [3.0 - 4.0]	0.706	**0.006**
	Overall image quality	2.5 [1.0 - 3.0]	2.0 [1.0 - 3.0]	0.914	**<0.001**

All data reported as median with interquartile ranges. *DS* Downsampled, *IM* Iodine map, *PURE* Lumen preserving spectral images, *UHR* Ultrahigh-resolution, *VMI* Virtual monoenergetic images.

Table S5 Subjective image quality rating for all stents and reconstructions at 80 beats per min

	Parameter	Reader 1	Reader 2	Weighted κ	*p*-value
UHR	Stent lumen visibility	3.0 [2.0 - 3.8]	3.0 [3.0 - 3.8]	0.767	**0.001**
	Strut delineation	3.0 [3.0 - 4.0]	3.0 [3.0 - 3.8]	0.531	**0.029**
	Artifact severity	2.0 [1.3 - 3.0]	2.5 [2.0 - 3.0]	0.706	**0.000**
	Overall image quality	3.0 [2.0 - 3.0]	3.0 [3.0 - 3.8]	0.634	**0.004**
VMI 45	Stent lumen visibility	3.0 [2.0 - 3.8]	3.0 [2.3 - 3.8]	0.340	0.121
	Strut delineation	2.0 [2.0 - 2.0]	2.0 [2.0 - 2.8]	-0.207	0.301
	Artifact severity	3.0 [2.3 - 3.0]	3.0 [2.0 - 3.0]	-0.087	0.778
	Overall image quality	2.5 [2.0 - 3.0]	3.0 [2.0 - 3.0]	0.474	**0.047**
VMI 55	Stent lumen visibility	3.0 [3.0 - 4.0]	3.5 [3.0 - 4.0]	0.231	0.289
	Strut delineation	2.5 [2.0 - 3.0]	2.0 [2.0 - 3.0]	0.333	0.197
	Artifact severity	3.0 [3.0 - 3.0]	3.0 [3.0 - 3.0]	0.231	0.236
	Overall image quality	3.0 [3.0 - 3.8]	3.5 [3.0 - 4.0]	0.605	**0.007**
VMI 70	Stent lumen visibility	3.0 [2.3 - 3.0]	3.0 [2.3 - 3.0]	0.265	0.253
	Strut delineation	3.0 [3.0 - 4.0]	3.0 [3.0 - 4.0]	0.762	**0.001**
	Artifact severity	3.0 [3.0 - 3.0]	3.0 [3.0 - 3.0]	0.286	0.236
	Overall image quality	3.0 [3.0 - 3.0]	3.0 [3.0 - 3.0]	0.615	**0.003**
VMI 85	Stent lumen visibility	2.0 [2.0 - 2.0]	2.0 [2.0 - 2.8]	0.167	0.490
	Strut delineation	3.0 [3.0 - 3.0]	2.5 [2.0 - 3.0]	0.565	**0.002**
	Artifact severity	3.0 [3.0 - 3.0]	3.0 [3.0 - 3.0]	0.474	**0.002**
	Overall image quality	2.5 [2.0 - 3.0]	3.0 [2.0 - 3.0]	-0.026	0.906
VMI 100	Stent lumen visibility	2.0 [1.0 - 2.0]	1.0 [1.0 - 2.0]	0.231	0.429
	Strut delineation	3.0 [2.3 - 3.0]	2.0 [1.3 - 2.0]	0.118	0.301
	Artifact severity	3.0 [2.0 - 3.0]	2.5 [2.0 - 3.0]	0.667	**0.010**
	Overall image quality	2.0 [1.3 - 2.0]	2.0 [2.0 - 2.8]	0.028	0.894
PURE	Stent lumen visibility	2.0 [2.0 - 3.0]	2.0 [2.0 - 3.0]	0.394	0.117
	Strut delineation	1.5 [1.0 - 2.0]	1.0 [1.0 - 1.8]	0.756	**0.003**
	Artifact severity	3.0 [2.0 - 3.0]	3.0 [2.3 - 3.0]	0.615	**0.022**
	Overall image quality	2.0 [2.0 - 3.0]	2.0 [2.0 - 2.0]	0.231	0.197
IM	Stent lumen visibility	3.0 [3.0 - 3.8]	3.5 [3.0 - 4.0]	0.500	**0.038**
	Strut delineation	2.0 [1.3 - 2.8]	2.0 [1.0 - 2.8]	0.659	**0.007**
	Artifact severity	3.0 [3.0 - 3.0]	4.0 [3.3 - 4.0]	0.048	0.707

	Overall image quality	3.0 [3.0 - 3.8]	3.5 [3.0 - 4.0]	0.600	**0.038**
DS	Stent lumen visibility	2.0 [1.0 - 2.8]	1.5 [1.0 - 2.8]	0.909	**0.000**
	Strut delineation	2.0 [1.0 - 2.0]	1.0 [1.0 - 1.8]	0.063	0.778
	Artifact severity	3.0 [2.0 - 3.0]	2.0 [2.0 - 3.0]	0.490	**0.008**
	Overall image quality	2.0 [1.3 - 3.0]	2.0 [1.0 - 3.0]	0.894	**0.001**

DS Downsampled, *IM* Iodine map, *PURE* Lumen preserving spectral images, *UHR* Ultrahigh-resolution, *VMI* Virtual monoenergetic images.

Table S6 Subjective image quality rating for all stents and reconstructions at 100 beats per min

	Parameter	Reader 1	Reader 2	Weighted κ	*p-value*
UHR	Stent lumen visibility	3.0 [2.0 - 3.8]	3.0 [2.3 - 4.0]	0.783	**0.002**
	Strut delineation	3.0 [3.0 - 3.8]	3.0 [3.0 - 3.0]	0.821	**0.001**
	Artifact severity	2.0 [2.0 - 3.0]	2.0 [2.0 - 2.8]	0.773	**0.001**
	Overall image quality	3.0 [2.0 - 3.0]	3.0 [2.3 - 3.8]	0.872	**0.001**
VMI 45	Stent lumen visibility	2.5 [2.0 - 3.0]	2.0 [2.0 - 3.0]	0.706	**0.001**
	Strut delineation	2.0 [2.0 - 2.0]	2.0 [2.0 - 2.0]	0.483	**0.030**
	Artifact severity	2.0 [2.0 - 2.0]	2.0 [2.0 - 2.0]	0.483	**0.030**
	Overall image quality	2.0 [2.0 - 3.0]	2.0 [2.0 - 3.0]	0.605	**0.006**
VMI 55	Stent lumen visibility	3.0 [3.0 - 4.0]	3.0 [2.3 - 3.8]	0.556	**0.004**
	Strut delineation	2.5 [2.0 - 3.0]	2.0 [2.0 - 3.0]	-0.286	0.197
	Artifact severity	2.0 [2.0 - 2.0]	3.0 [2.0 - 3.0]	-0.053	0.747
	Overall image quality	3.0 [2.3 - 3.8]	3.0 [3.0 - 3.0]	0.118	0.490
VMI 70	Stent lumen visibility	3.0 [2.3 - 3.8]	3.0 [2.3 - 3.0]	0.333	0.111
	Strut delineation	3.0 [3.0 - 3.0]	2.0 [2.0 - 2.0]	0.000	>0.999
	Artifact severity	2.0 [2.0 - 2.0]	3.0 [2.0 - 3.0]	0.118	0.389
	Overall image quality	3.0 [2.0 - 3.0]	3.0 [3.0 - 3.0]	-0.176	0.328
VMI 85	Stent lumen visibility	2.0 [2.0 - 2.8]	2.0 [1.0 - 3.0]	0.186	0.260
	Strut delineation	3.0 [3.0 - 3.0]	2.0 [2.0 - 2.0]	0.074	0.429
	Artifact severity	2.0 [2.0 - 2.8]	2.0 [2.0 - 3.0]	0.242	0.260
	Overall image quality	2.0 [2.0 - 2.8]	2.0 [2.0 - 3.0]	-0.081	0.701
VMI 100	Stent lumen visibility	2.0 [1.0 - 2.0]	1.0 [1.0 - 2.0]	0.194	0.429
	Strut delineation	3.0 [2.3 - 3.0]	1.5 [1.0 - 2.0]	0.000	>0.999
	Artifact severity	3.0 [2.0 - 3.0]	2.0 [2.0 - 2.8]	0.054	0.778
	Overall image quality	2.0 [1.3 - 2.0]	2.0 [2.0 - 2.0]	0.167	0.395
PURE	Stent lumen visibility	2.0 [2.0 - 2.8]	2.0 [1.0 - 3.0]	0.318	0.121
	Strut delineation	1.5 [1.0 - 2.8]	1.0 [1.0 - 1.8]	0.375	**0.038**
	Artifact severity	2.5 [2.0 - 3.0]	3.0 [2.0 - 3.0]	0.286	0.197
	Overall image quality	2.0 [1.3 - 2.8]	2.5 [2.0 - 3.0]	0.375	**0.038**
IM	Stent lumen visibility	3.0 [3.0 - 3.8]	4.0 [3.0 - 4.0]	0.375	0.091
	Strut delineation	2.0 [1.3 - 2.8]	1.0 [1.0 - 1.8]	0.205	0.175
	Artifact severity	3.0 [2.3 - 3.0]	3.0 [3.0 - 4.0]	0.000	>0.999

	Overall image quality	3.0 [3.0 - 3.0]	3.0 [3.0 - 4.0]	0.375	0.053
DS	Stent lumen visibility	2.0 [1.0 - 2.0]	2.0 [1.0 - 2.0]	0.222	0.307
	Strut delineation	1.0 [1.0 - 2.0]	1.0 [1.0 - 1.0]	0.231	0.197
	Artifact severity	3.0 [2.3 - 3.0]	2.0 [2.0 - 3.0]	0.268	0.169
	Overall image quality	1.5 [1.0 - 2.0]	2.0 [1.3 - 2.0]	0.512	0.027

DS Downsampled, *IM* Iodine map, *PURE* Lumen preserving spectral images, *UHR* Ultrahigh-resolution, *VMI* Virtual monoenergetic images.

Table S7 Results of Friedman's test followed by Wilcoxon signed rank test for each reader between all heart rates

Name	Reconstruction	Friedman	60 vs 80 bpm	60 vs 100 bpm	80 vs 100 bpm
**Reader 1**					
Stent lumen visibility	UHR	**0.002**	**0.011**	**0.011**	1.000
	VMI 45	0.232	1.000	0.257	0.083
	VMI 55	0.717	0.564	0.564	1.000
	VMI 70	0.091	0.059	0.257	0.157
	VMI 85	0.607	0.317	1.000	0.317
	VMI 100	1.000	1.000	1.000	1.000
	PURE	0.472	0.564	0.317	0.317
	IM	**0.002**	**0.014**	**0.014**	1.000
	DS	0.819	0.705	0.414	0.564
Strut Delineation	UHR	**0.006**	**0.034**	**0.020**	0.317
	VMI 45	0.368	0.317	0.317	1.000
	VMI 55	1.000	1.000	1.000	1.000
	VMI 70	0.819	1.000	0.564	0.564
	VMI 85	0.717	0.564	0.564	1.000
	VMI 100	**0.039**	0.083	**0.046**	0.317
	PURE	0.717	0.655	0.414	0.317
	IM	0.368	0.317	0.317	1.000
	DS	0.135	1.000	0.157	0.157
Artifact severity	UHR	**0.000**	**0.006**	**0.004**	1.000
	VMI 45	**0.000**	**0.008**	**0.006**	**0.008**
	VMI 55	**0.000**	**0.008**	**0.006**	**0.008**
	VMI 70	**0.000**	**0.003**	**0.004**	**0.008**
	VMI 85	**0.001**	**0.046**	**0.005**	**0.008**
	VMI 100	**0.003**	**0.008**	**0.020**	1.000
	PURE	**0.004**	**0.024**	**0.015**	0.564
	IM	0.152	0.527	0.132	0.083
	DS	**0.016**	**0.038**	**0.038**	1.000
Overall Image Quality	UHR	**0.001**	**0.008**	**0.008**	1.000
	VMI 45	**0.015**	**0.046**	**0.034**	0.157
	VMI 55	0.472	0.564	0.317	0.317
	VMI 70	0.066	0.083	0.059	0.317
	VMI 85	**0.015**	**0.046**	**0.034**	0.157
	VMI 100	**0.007**	**0.034**	**0.034**	1.000
	PURE	0.050	0.157	0.059	0.180
	IM	0.092	0.564	**0.046**	0.180
	DS	0.179	0.739	0.096	0.157
**Reader 2**					
Stent lumen visibility	UHR	**0.013**	**0.023**	**0.038**	1.000
	VMI 45	0.060	0.234	**0.024**	0.160
	VMI 55	0.223	0.527	0.059	0.340
	VMI 70	0.124	0.059	0.096	0.739
	VMI 85	**0.029**	**0.046**	**0.034**	0.317
	VMI 100	**0.015**	**0.025**	**0.046**	0.317
	PURE	**0.040**	0.059	**0.046**	0.180
	IM	0.368	0.180	0.414	0.564
	DS	0.280	0.257	0.102	0.655
Strut delineation	UHR	0.141	0.102	0.096	0.655
	VMI 45	0.055	0.180	**0.038**	0.157
	VMI 55	0.554	0.414	0.257	0.655
	VMI 70	**0.028**	0.114	0.317	**0.031**
	VMI 85	**0.043**	0.096	0.564	**0.034**
	VMI 100	0.311	0.480	0.317	0.102
	PURE	0.082	0.102	0.157	0.157
	IM	**0.035**	0.157	0.414	**0.014**
	DS	0.607	0.655	0.564	0.317
	UHR	**0.000**	**0.006**	**0.006**	0.180
	VMI 45	**0.001**	**0.013**	**0.006**	0.059
	VMI 55	**0.012**	**0.035**	**0.015**	0.317
	VMI 70	**0.001**	**0.007**	**0.005**	0.317
Artifact severity	VMI 85	**0.002**	**0.025**	**0.009**	**0.034**

	VMI 100	0.179	0.234	0.068	0.180
	PURE	**0.013**	**0.023**	**0.038**	0.705
	IM	0.289	0.414	0.102	0.480
	DS	**0.001**	**0.006**	**0.008**	0.655
Overall Image Quality	UHR	**0.002**	**0.023**	**0.015**	0.157
	VMI 45	**0.005**	0.084	**0.009**	0.059
	VMI 55	0.449	1.000	0.157	0.317
	VMI 70	**0.041**	**0.046**	**0.046**	1.000
	VMI 85	0.054	0.317	**0.020**	0.157
	VMI 100	0.646	0.480	0.317	1.000
	PURE	0.074	0.083	0.564	0.046
	IM	0.074	0.180	**0.046**	0.317
	DS	0.727	0.705	0.480	0.739

DS Downsampled, *IM* Iodine map, *PURE* Lumen preserving spectral images, *UHR* Ultrahigh-resolution, *VMI* Virtual monoenergetic images.
